# Flexible Drinking Straws of the Cretaceous: A Novel Type of Labium in Mesozoic Auchenorrhyncha and Its Functional Implications

**DOI:** 10.3390/insects17080789

**Published:** 2026-07-29

**Authors:** Dolev Fabrikant, Yanzhe Fu, Leonidas-Romanos Davranoglou

**Affiliations:** 1 The Steinhardt Museum of Natural History, Tel Aviv University, 12 Klausner Street, Tel Aviv 6997801, Israel; dolevf@mail.tau.ac.il; 2 New Environmental School, Tel Aviv University, Tel Aviv 6100000, Israel; 3 State Key Laboratory of Palaeobiology and Stratigraphy, Nanjing Institute of Geology and Palaeontology, Chinese Academy of Sciences, Nanjing 210008, China; yzfu@nigpas.ac.cn

**Keywords:** Fulgoromorpha, Cretaceous, planthoppers, Hemiptera, amber, morphology, mouthparts

## Abstract

The evolution of novel feeding structures played a major role in the diversification of Hemiptera. Here, we investigate the morphology and inferred function of a highly specialized labium in the extinct Cretaceous planthopper families Neazoniidae and Mimarachnidae. These insects possessed a feeding apparatus unparalleled among extant Hemiptera that is characterized by an extremely elongated labium with a densely corrugated apical segment formed by sinusoidal cuticular folds. Comparative analysis, aided by micro-computed tomography, indicates that the cuticular corrugations were a mechanical adaptation that increased the flexibility of the tubular feeding structure while resisting kinking and buckling. We propose that this morphology facilitated probing within deep bark fissures and other mechanically resistant plant tissues, potentially enabling access to the vascular bundles. Similar corrugated feeding structures convergently occur in several unrelated Cretaceous hemipterans as well as some long-proboscid Mecoptera, suggesting that extinct Mesozoic ecosystems supported feeding strategies absent in their modern relatives. Furthermore, we propose the synonymy of Mimarachnidae with Neazoniidae based on the shared possession of this specialized labium morphology, along with other morphological features, establishing the taxonomic connection between nymph and adult specimens. We also transfer the unplaced genus *Spinonympha* to the family Neazoniidae and describe a new species of the genus *Cretodorus*.

## Simple Summary

Planthoppers (Fulgoromorpha) feed using straw-like mouthparts that pierce plant tissues and extract nutrient-rich internal fluids. Modifications of these feeding structures have contributed substantially to the evolution and diversification of the group. This study examines extinct planthoppers preserved in amber from approximately 100 million years ago that possessed a remarkable feeding apparatus unlike that of any living or extinct relatives. Their elongated mouthparts, often exceeding the length of the body, terminated in a segment covered in fine, accordion-like folds whose function has remained unclear. By comparing the fine mouthpart morphology using micro-computed tomography (a high-resolution 3D X-ray imaging technique) we found that the cuticular folds likely represent a mechanical adaptation, increasing flexibility while resisting kinking or collapse, much like a bendy drinking straw or a flexible vacuum hose. We propose that this adaptation enabled the insects to probe deep cracks and fissures in bark to reach internal plant fluids inaccessible by more rigid mouthparts. We also observed that the mouthpart tip could expand like a wall plug and may have helped with anchoring to the surface during feeding. Similar corrugated structures evolved independently in several unrelated Cretaceous insects, suggesting that these ancient forests supported feeding strategies lacking close analogues among living taxa.

## 1. Introduction

Understanding how organisms evolve novel morphological structures to exploit ecological opportunities is a central question in biology, with implications for ecology, developmental biology, and evolutionary theory [1]. Insects, with their extraordinary diversity and morphological plasticity, offer unparalleled insight into this process [2]. Among them, Hemiptera represent one of the most ecologically and morphologically diverse insect orders, comprising over 110,000 described species (roughly 7% of all Metazoa [3]) which occupy nearly every terrestrial and freshwater habitat, including the ocean’s surface [4]. They also include important agricultural and medical pests, as well as groups of significant commercial interest [5]. Furthermore, Hemiptera are among the most ancient insect groups, first appearing in the Late Carboniferous, more than 320 million years ago [6,7,8].

The longstanding success of this megadiverse group is attributed in part to the morphologically distinct piercing-sucking mouthparts that enabled the development of novel feeding strategies and the exploitation of previously unutilized food sources [9,10]. The hypodermic-needle-like hemipteran mouthparts are composed of the labium, which is a highly derived structure that functions as a sheath for internal, rapier-like structures known as the stylets; the latter pierce tissues and enable fluid feeding [4,11,12]. The labium has allowed Hemiptera to develop a diverse range of feeding strategies, including carnivory, haematophagy, granivory, fungivory, and especially sucking plant saps, more so than any other group of insects [13,14].

Fulgoromorpha (Hemiptera: Auchenorrhyncha), also known as planthoppers, are a cosmopolitan infraorder that comprises more than 13,500 described species in 22 extant and 18 extinct families [15], with an origin dating to the Permian (263 million years ago, mya) or the Carboniferous (330 mya) [16]. Several species are important crop pests [17]. Fulgoromorpha are characterized by their posteriorly oriented labium, ventrally inserted antennae that possess a setiform distal segment (flagellum) and an enlarged base (pedicel) with sensory plaques, presence of specialized sensory pits in nymphs, a Y-shaped venation on the tegmina clavus, fusion of the metacoxae to the thoracic sternites, reduced setation on the metatibia, and a special mode of abdominal vibration known as the snapping organ [18,19,20,21]. This group of insects is almost exclusively phytophagous and often displays tight associations with specific host plants on which they spend most of their life feeding [14,22,23,24].

The appearance of extraordinary insect morphologies during the Early Cretaceous is well known and has been associated with the biological turnover occurring in ecosystems during the Terrestrial Mesozoic Revolution [25]. The Early Cretaceous coincided with a peak in the taxonomic radiation of Hemiptera and the development of morphologically aberrant forms with no parallels in present-day hemipterans [10,26]. Two extinct, morphologically aberrant planthopper families from the Cretaceous, the Neazoniidae Szwedo, 2007 and Mimarachnidae Shcherbakov, 2007, also fall within this pattern. Neazoniidae are represented by three species from the Early Cretaceous Archingeay (France) and Lebanese ambers and are known exclusively from nymphs [27,28,29]. Mimarachnidae, on the other hand, are primarily known from adult specimens and currently comprise 25 described species that display a wide temporal and geographic distribution across the Early Cretaceous of Eurasia [30,31]. These families are distinguished exclusively by the distribution and positional patterns of sensory pits, and previous studies have suggested that they might be closely related or even synonymous [32,33,34,35]. Indeed, the nymphs of two families are morphologically very similar and are both characterized by an elongated labium with a uniquely corrugated (annulated) surface [27,28,30].

**Figure 1:**
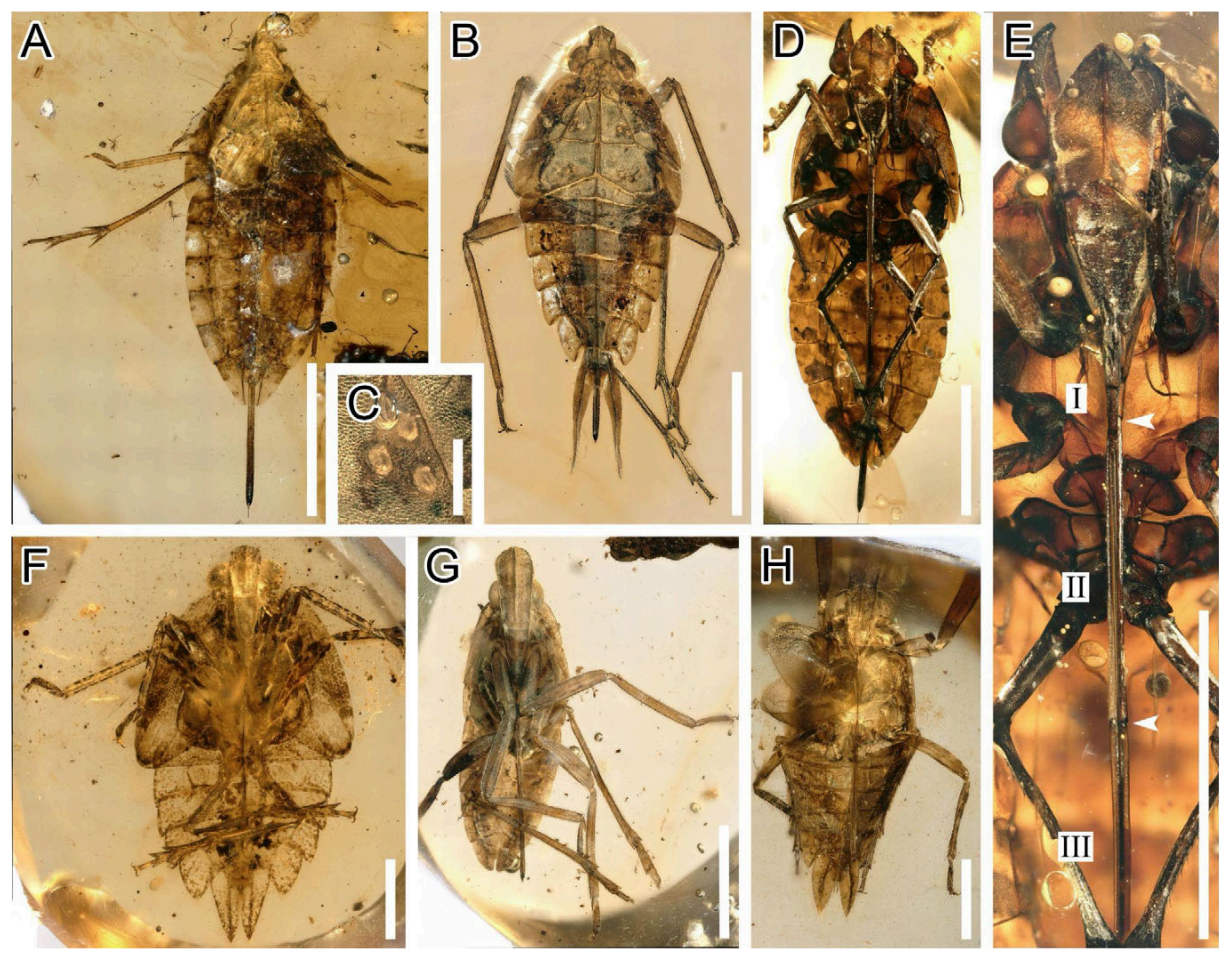
Overview of examined Neazoniidae nymphs exhibiting marked morphological disparity in body proportions and labium length. Scale bars: 2 mm, Scale (**C**): 200 μm (**A**) NIGP210709: Dorsal view. (**B**) NIGP210710: Dorsal view. (**C**) Same, magnification of sensory pit rosette on mesonotum. (**D**) NIGP210711: Ventral view. (**E**) Same, magnified view of head and labium, white arrows pointing to segment joints; segments I–III are numbered. (**F**) NIGP210712: Ventral view. (**G**) NIGP210713: Ventral view. (**H**) Specimen NIGP210714: Ventral view.

**Figure 2:**
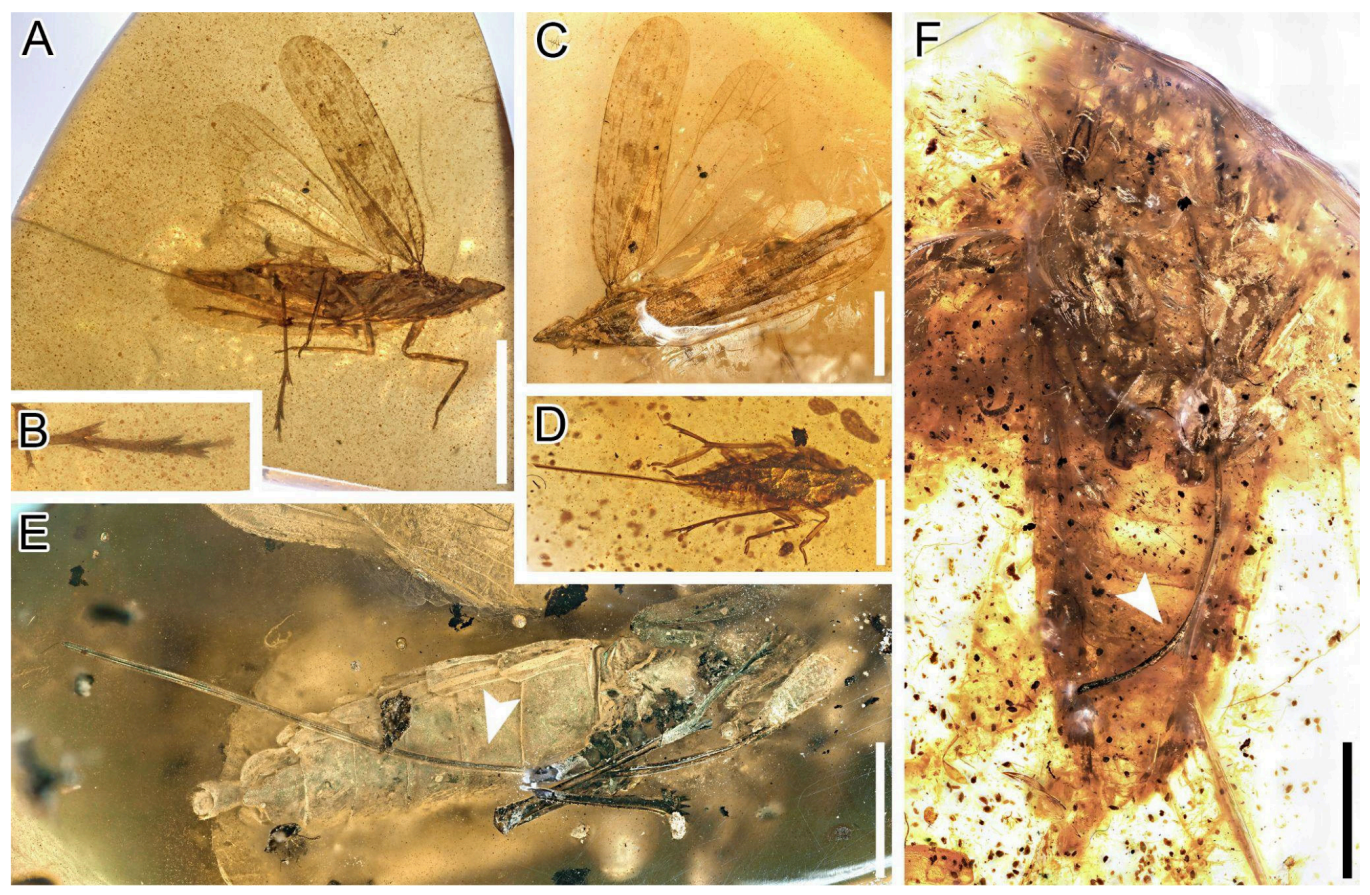
Overview of *Cretodorus noonoo* sp. nov. and Neazoniidae specimens displaying labia preserved in curved conformations. Scale bars: 2 mm, Scale (**C**): 1 mm. (**A**) *C. noonoo* sp. nov. Holotype (NIGP210707), ventral habitus. (**B**) Same, magnification of right metatibia and metarasus (**C**) Same, dorsal habitus. (**D**) *C. noonoo* sp. nov. paratype (NIGP210716), dorsal habitus. (**E**) *Cretodorus* sp. (NIGP210706), showing distal labium segment flexing dorsoventrally (see arrow). (**F**) Neazoniidae indet. (NIGP210715), showing distal labium segment flexing dorsoventrally (see arrow).

Previous studies on these planthoppers noted the consistent elongation of the labium, however its detailed morphology was never described and the distinctly corrugated surface has not been mentioned or described, despite appearing in illustrations ([30] Figure 5B p. 242; [36] Figure 2B p. 4; [27] Figure 9 p. 130, Figure 21 p. 133; [28] Figure 1F p. 108). Despite this paucity of morphological information, the elongated labium has been described as an adaptation for feeding on the thicker branches of woody plants [28,32,36,37]. However, inferring function in the absence of anatomical data results in a significant knowledge gap, as mouthpart morphology provides important insights into ecology since feeding structures are shaped by dietary specialization and mediate direct interactions with the environment. Form and structural organization determine their mechanical performance and are consequently associated with the utilization of different food resources between species [11,38,39,40,41].

In this study we directly examine a selection of adult and nymphal specimens of Mimarachnidae preserved in Cenomanian amber to document the structural and morphological details of the labium and draw comparisons to the morphology of Neazoniidae as recorded in the scientific literature. Further, we employ synchrotron-based X-ray micro computed tomography (SR-micro-CT) to investigate the internal and cross-sectional anatomy of the labial cuticle and stylets. Our findings reveal that the labium of neazoniid and mimarachnid planthoppers has been modified into a highly specialized feeding structure that lacks analogues among currently known living or extinct Auchenorrhyncha.

## 2. Materials and Methods

We implement the terminology of fulgoromorphan wing venation as specified by Bourgoin et al. [42] and the established interpretation of wing venation in Mimarachnidae [29]. The recognition of the derived structure of the labium allowed us to provide further support for the assignment of the commonly occurring planthopper nymphs in Burmese amber to Mimarachnidae [30,32,36].

### 2.1. Macrophotography

The amber pieces analyzed in this study were polished using sandpaper at increasingly finer grades and finished with high grade polishing paste. In preparation for imaging the specimens were mounted on slides using adhesive putty, the surface was dripped with water and covered in a microscope slide for clearer visibility. Photographs were taken using a Keyence VHX-6000 (Keyence, Osaka, Japan) digital microscope and using Keyence BZ X810 for fluorescence images. The resulting photographs were further focus stacked using Helicon Focus Pro 8.2.2 (Helicon Soft, Kharkiv, Ukraine) and processed in Adobe Photoshop CC 2019 (Adobe, San José, CA, USA) to adjust contrast and brightness.

### 2.2. Synchrotron X-Ray Micro-Computed Tomography

X-ray tomographic microscopy scans of a critical point dried male specimen of an Issidae from Mozambique were obtained at the TOMCAT beamline, Swiss Light Source (SLS), Paul Scherrer Institut, Switzerland, at an X-ray beam energy of 15.99 keV with final pixel size of 1.625 μm. A fossil specimen of an adult Mimarachnidae (NIGP210705) was scanned at the P05 imaging beamline of PETRA III, DESY (Deutsches Elektronen-Synchrotron, Hamburg, Germany), using propagation phase-contrast synchrotron radiation micro-computed tomography [43]. Scans were performed at an X-ray beam energy of 19.997 keV with an effective pixel size of 0.664 µm and a binned pixel size of 1.329 µm. Three-dimensional image processing and segmentation was carried out using Amira 3D version 2025.1 software (Mercury Systems, Andover, MA, USA). Image labelling and illustrations were generated in Adobe Illustrator CC/CS6 (Adobe Systems Incorporated, San Jose, CA, USA).

### 2.3. Materials 

The material examined in this study includes one modern specimen deposited at the Oxford University Museum of Natural History under the repository number OUMNH MZ8, and thirteen fossil specimens preserved as inclusions in Early Cenomanian age Kachin amber (Myanmar), which are deposited in the Nanjing Institute of Geology and Palaeontology under the repository numbers NIGP210705, 210706, 210707, 210708, 210709, 210710, 210711, 210712, 210713, 210714, 210715, 210716, and 210717.

## 3. Results

### 3.1. Detailed Morphological Description of the Mouthparts of Neazoniidae and Mimarachnidae

#### 3.1.1. Labium

**Figure 3:**
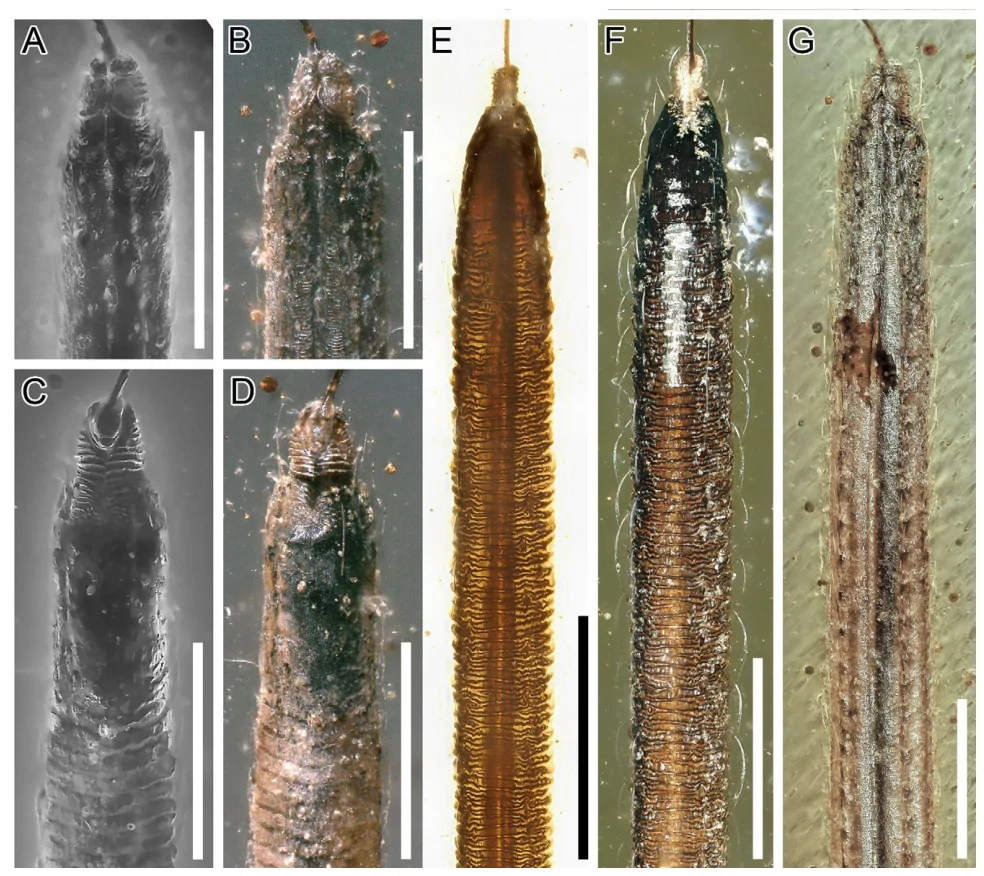
Detailed morphology of the third labial segment in nymphal and adult specimens of Neazoniidae. Scale bars: 200 μm. (**A**) *Cretodorus* sp. (NIGP210706), High magnification of labium apex, fluorescence photograph in dorsal view, (**B**) Same, reflected light photograph. (**C**) Same, Texas Red fluorescence photograph in ventral view. (**D**) Same, white light photograph in ventral view. (**E**) Neazoniidae indet. (NIGP210709), transmitted light photograph of distal labium segment in ventral view. (**F**) Same, reflected light photograph. (**G**) (NIGP210706), reflected light photograph of distal labium segment in dorsal view.

**Figure 4:**
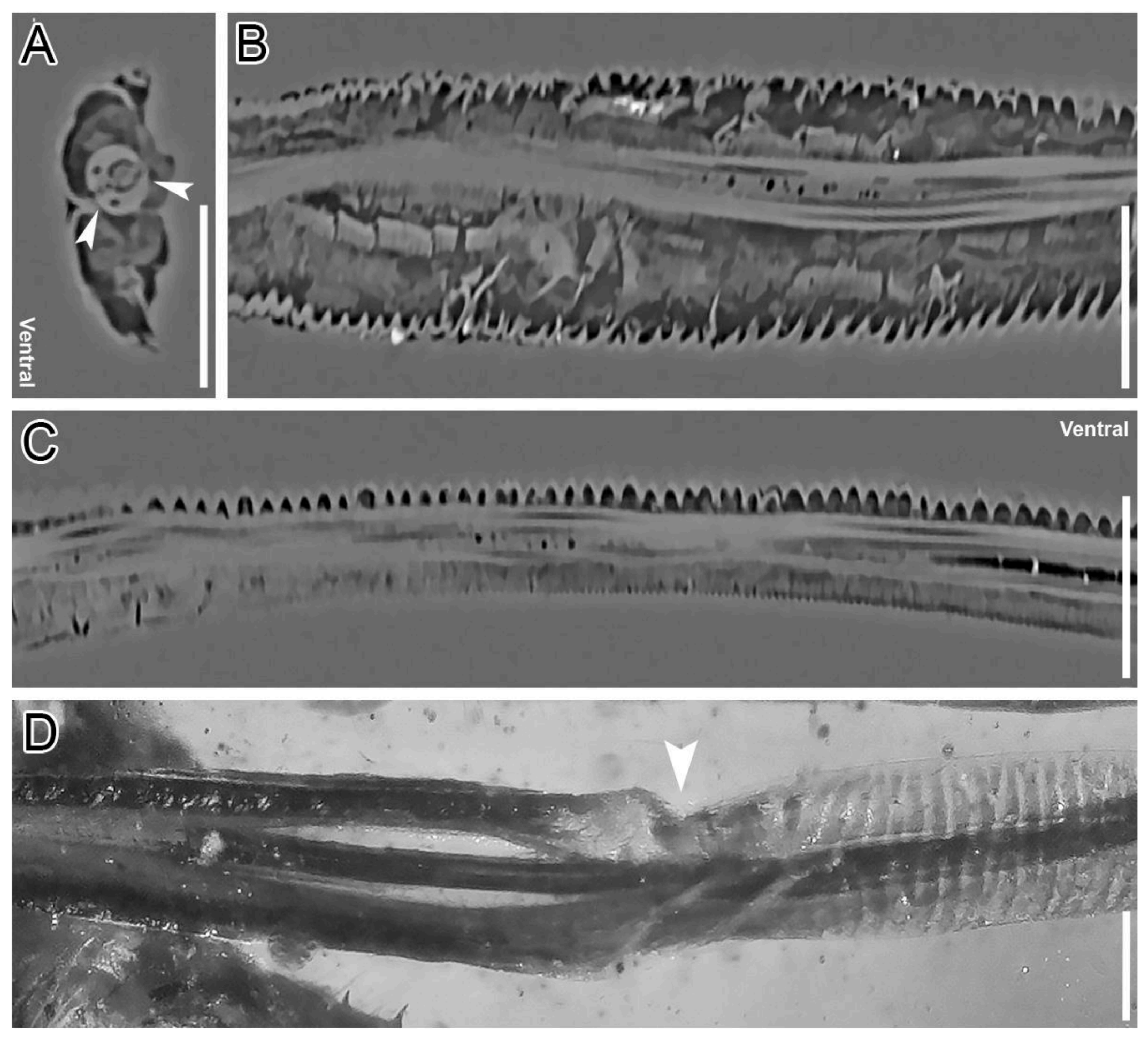
Photographs and micro-CT slices showing the detailed structure of the third labium segment of Neazoniidae. Scale bars: 100 μm. (**A**) *Burmissus* sp. (NIGP210705), Micro-CT section in transverse view showing two pairs of stylets (arrows pointing to dorsal and ventral mandibular tips). (**B**) Same, in horizontal section showing lateral corrugations. (**C**) Same, sagittal section showing sinusoid corrugated cuticle on ventral surface and smooth cuticle on dorsal surface. (**D**) *Cretodorus* sp. (NIGP210708), magnified view of the elbow-like articulation between the second and third labium segments (see arrow), showing transition between smooth to corrugated sections, dorsal view.

The adults and nymphs of all examined Neazoniidae and Mimarachnidae where the head is preserved possess a significantly elongated labium that can often be seen emerging from the posterior margin of the insect at rest (Figure 1A–D,G). In some specimens the labium reaches the metacoxae while in others it is so long that it exceeds twice the body length (Figure 1F and Figure 2A–C). Despite its extreme elongation, the labium of both groups displays the standard three-segmented division of Fulgoromorpha, with the apical segment being the most elongated and variable between taxa (Figure 1) [20]. However, in addition to this feature, uniquely among Auchenorrhyncha, the apical segment (III) of the labium is densely covered in uniform transverse corrugations on its ventral and lateral surfaces (Figure 3C–F). These corrugations appear to have conferred flexibility, as in several specimens the labium is preserved in a curved or bent position (Figure 2D,E). The articulation between the second and third labial segments is distinctly elbow-like and may have enhanced mobility of the third segment by facilitating the tilting into anterior positions (Figure 4D). Computed tomographic scanning shows that the cuticle of the apical labial segment of Neazoniidae and Mimarachnidae is uniform in thickness and the corrugated surface sculpture is formed by sinusoid folds of the cuticle (Figure 4). It is likely that the regular folds on the labium are an adaptation that increased the flexibility and durability of this structure (see Section 4.2).

The dorsal surface of the labium is smooth and divided by a deep longitudinal groove which encloses the stylet bundle (Figure 3A,B,G, and Figure 4D). The cuticle on the margins of the labial groove is ornamented with very shallow irregular striations (Figure 3B,G). Laterally, the surface of the dorsal cuticle is smooth and studded on either side by two rows of long setae which possibly served a sensory function (Figure 3F,G).

The apex of the labium is darkened, and its tip is modified into a projecting ribbed, segment-like structure (Figure 3). Cuticle darkening is a common trait associated with increased sclerotization through protein crosslinking [44]. Such sclerotization is common in Fulgoromorpha and helps to protect the labium apex and associated sensory structures from prolonged contact with the plant surface during feeding. The presence of structural reinforcement in Mimarachnidae is supported by the observation that the apex of the third labial segment remains three dimensional even in specimens that have undergone significant taphonomic deformation or crushing whereas the proximal, lighter colored, areas are typically fully flattened (personal observation by D. Fabrikant).

The ribbed protruding tip of the labium is divided into two lateral lobes bearing transverse serrations (Figure 3A–D). These lobes are separated by a median seam visible on both the ventral and dorsal surfaces. Dorsally the structure is divided into four smaller sections which are demarcated by transverse furrows (Figure 3A,B).

In a closed orientation, the tip structure appears to have been narrower than the complete stylet bundle (Figure 3E). Considering the dimensions of the protruding labium tip, the simultaneous emergence of the mandibular and maxillary stylets during feeding would have forced the structure to expand and the lateral lobes to open (see Section 4.3). The range of mobility of this structure is hard to estimate and its functionality remains elusive, however it possibly served to anchor the labium to the surface.

#### 3.1.2. Stylet Bundle

**Figure 5:**
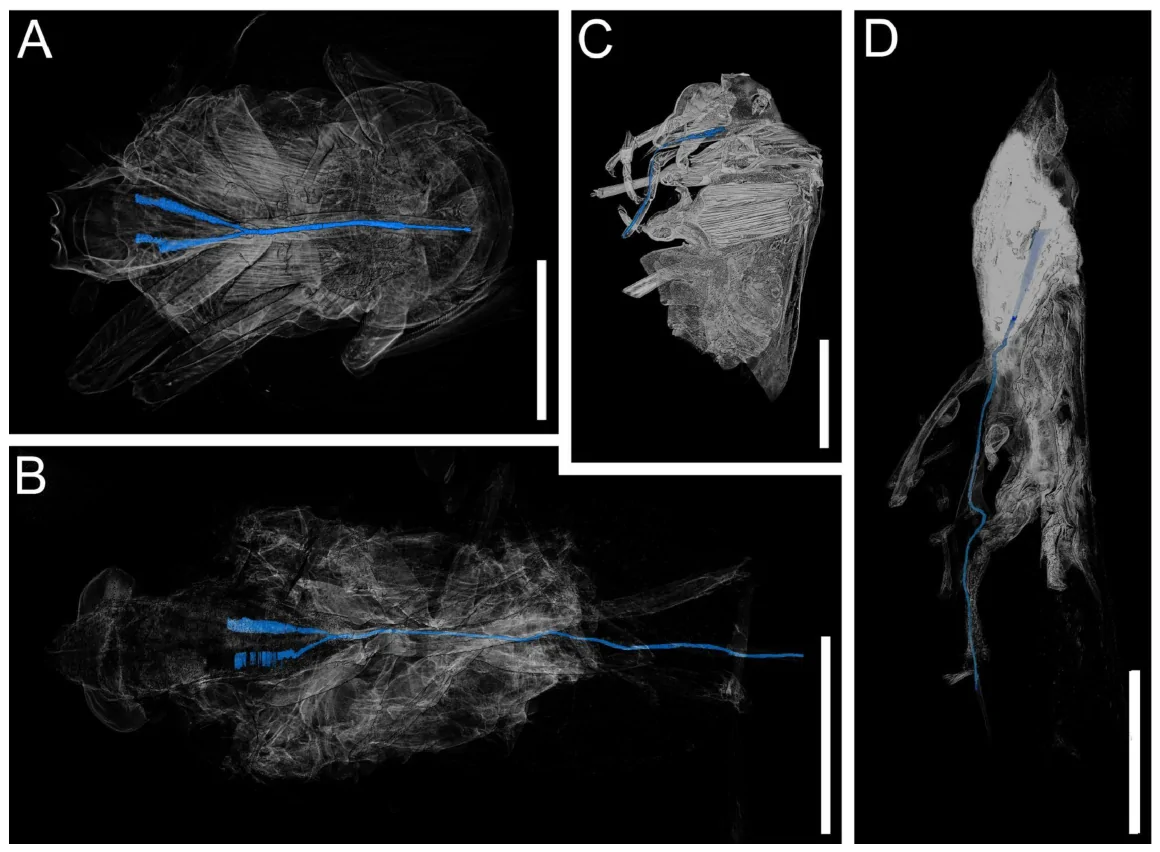
Micro-CT renderings comparing general habitus and stylet (blue) morphology in extant Issidae indet. and Neazoniidae. Scale bars: 2 mm. (**A**) Issidae indet. (OUMNH-MZ8), in horizontal view (**B**) *Burmissus* sp. (NIGP210705), in horizontal view. (**C**) Issidae indet. (OUMNH-MZ8), in sagittal section (**D**) *Burmissus* sp. (NIGP210705), in sagittal section.

The stylet bundle is much longer than extant forms, as its length is correlated to the unusually long labium in this group of insects (Figure 5). In the cross section, the stylet bundle appears standard for Fulgoromorpha (Figure 4A). The mandibular dorsal tip appears to be tapering, and the mandibular ventral tip appears to be wide and flattened (Figure 4A). This condition is shared with Caliscelidae, Delphacidae, Derbidae and Issidae [45]. The maxillae are almost entirely surrounded by the mandibles, except for a narrow portion near the dorsal margin which remains exposed in a condition similar to most Fulgoromorpha except for Cixiidae, Issidae and Lophopidae where the maxillae were observed to be fully enveloped by the mandibles [45]. Overall, in subapical cross-section the stylet bundle of Mimarachnidae appears to be very similar in proportion to Delphacidae ([45] Figure 4 p. 244). However, these similarities should not be taken as evidence for phylogenetic relationships since the detailed morphology of the labium and distribution of the fine morphological characters is unknown for most families of Fulgoromorpha [45].

### 3.2. Systematic Section

#### 3.2.1. Synonymy of Mimarachnidae with Neazoniidae

The nymphs of Mimarachnidae are almost indistinguishable from Neazoniidae. The distinction between Neazoniidae and Mimarachnidae has historically relied primarily on differences in the arrangement and distribution of sensory pits on the body [27,28,29]. However, comparison of the currently available material suggests that these differences are insufficient to justify separation at family level. Indeed, previous studies have also suggested a close relationship between the two groups and even raised the possibility that they may represent synonymous taxa [32].

The previously proposed affiliation between Neazoniidae with Mimarachnidae is supported by several shared derived morphological characters. Most notably, both groups possess an extremely elongated labium with a uniquely corrugated or annulated distal segment (Figure 3), a condition unknown in other extinct or extant Fulgoromorpha [27,28]. The detailed structure of the labium, including the distribution of corrugation on the lateral and ventral surfaces and the apparent flexibility of the apical segment, is identical in both groups ([30] Figure 5B p. 242; [36] Figure 2B p. 4; [27] Figure 9 p. 130, Figure 21 p. 133; [28] Figure 1F p. 108). Given the structural complexity and apparent functional specialization of this morphology, its independent evolution in two otherwise highly similar groups would appear non-parsimonious.

Additional similarities include a reduction of metatibial armature, flattened body form, and similar body proportions [27,32,35]. In both groups, sensory pits occur in repeated clusters and display similar placement on the thorax and abdomen [30]. The nymphs of Neazoniidae and Mimarachnidae are morphologically very similar and their distinction has been repeatedly questioned in light of the increased availability of immature specimens in Kachin amber [32,34,35].

The difficulty in assessing the question on the relationship between the two families is hampered by the incompleteness of the fossil record. Neazoniidae are known exclusively from nymphs, whereas Mimarachnidae were originally described primarily on the basis of adults and wing venation characters [28,29]. Only recently have studies begun attempting to associate nymphal morphotypes with adult mimarachnids preserved in Burmese amber [30,32].

The principal character historically separating the two families involves the arrangement of sensory pits. In Neazoniidae, pits are frequently arranged in rosette-like groupings composed of triplets or quadruplets, whereas in Mimarachnidae the pits are generally more dispersed and commonly occur in pairs. However, exceptions to these patterns occur in both groups, including distinct three-part rosettes on the mesonotum of some mimarachnid nymphs and adults (Figure 1C) [30]; while some specimens of both groups possess only single pits in places like the abdominal tergites where other specimens display clear pit clusters (Figure 1A,D; [28] Figure 1A p. 108).

Although useful as a taxonomic character, sensory pit arrangement has been documented to vary considerably among fulgoromorphan taxa and especially between developmental stages even within a species [18]. Intrafamilial variability of sensory pit structure, grouping, and placement is exemplified by the following (non-exhaustive) instances: the family Dictyopharidae includes genera both presenting (*Dictyophara*) and lacking (*Nymphorgerius*, *Haumavarga*) sensory pits on the laterotergites [46]; Nogodinidae and Issidae species vary in the presence of sensory pits on the pregenital sternites [47,48]; some species of Ricaniidae possess triads of basally connected sensory pits [46], while others lack them [49]; in Caliscelidae, the Adenissini tribe is distinguished from other representatives of the family by closely connected sensory pits on the pronotum [50]. Given the high variability of these structures in extant Fulgoromorpha and among Neazoniidae, the relatively minor differences observed between Neazoniidae and Mimarachnidae do not appear taxonomically sufficient to support family-level separation.

The recognition of the distinctive corrugated labium in both groups substantially strengthens the argument for synonymy because it represents a highly specialized and previously overlooked morphological character system. The consistent occurrence of this structure in both nymphs attributed to Neazoniidae and adults attributed to Mimarachnidae provides additional support for the interpretation that these taxa belong to a single evolutionary lineage. We therefore consider Mimarachnidae to represent a junior synonym of Neazoniidae and provide an amended diagnosis for the family.

Suborder Auchenorrhyncha Duméril, 1805

Infraorder Fulgoromorpha Evans, 1946

Superfamily Fulgoroidea Kirkaldy, 1907

Family Neazoniidae Szwedo, 2007 (=Mimarachnidae Shcherbakov, 2007 syn. nov.)

Emended Diagnosis: Neazoniidae can be identified by the following combination of characters: head triangular with well-developed lateral carinae; labium long, segment III longest and covered in transverse corrugations; apex of labium terminating in bilobed ribbed structure; pronotum and mesonotum with double median carinae; tegminal venation simplified, lacking distinct crossveins separate from the irregularly distributed polygonal meshwork of delicate veinlets; single prenodal CuA fork; basal cell absent or weakly delimited; clavus open; hindwing with stem M single, with meshwork of veinlets and multiple veins on anal field; forelegs and midlegs with two visible tarsomeres; metatibia lacking longitudinal armature except for basal spine in some species; metatibial pecten bearing 4–8 teeth; metatarsal pectens bearing 4–8 teeth. The body structure of neazoniid nymphs is similar, except for the following characters: dorsoventrally flattened body with at least some sensory pits arranged in clusters of two to four with inwardly pointing setae.

Remarks: Previously, sensory pit persistence in adults was described as a diagnostic character, however this trait is only known from the Genus *Cretodorus* and thus was not included in the emended diagnosis [30,33]. Additionally, Neazoniidae lack clear crossveins that are separate from the irregular meshwork of veinlets. The only reported exception to this rule is *Tenebricusus* He, Jiang and Szwedo, 2022, however a re-examination of the specimen has revealed that the crossvein between M and CuA veins is illusory and actually represents a crack in the amber matrix [51].

A setigerous pecten was initially described by Shcherbakov [29] and Szwedo & Ansorge [37] as diagnostic for Mimarachnidae but was later amended as a variable trait in light of new specimens from Kachin amber and was therefore not included in the emended diagnosis [52].

#### 3.2.2. Transfer of *Spinonympha shcherbakovi* Luo et al., 2021 to Neazoniidae

Suborder Auchenorrhyncha Duméril, 1805

Infraorder Fulgoromorpha Evans, 1946

Superfamily Fulgoroidea Kirkaldy, 1907

Family Neazoniidae Szwedo, 2007 (=Mimarachnidae Shcherbakov, 2007 syn. nov.)

*Spinonympha shcherbakovi* Luo et al., 2021

Previously, *S. shcherbakovi* had not been assigned to any known fulgoromorphan family due to the general absence of clear diagnostic characters such as wing venation, and the poor state of preservation of the specimen which obscures the arrangement of sensory pits on the thorax [36].

*S. shcherbakovi* can be assigned to Neazoniidae based on the presence of a flexible elongated distal segment of the labium displaying transverse annulations with a modified protruding structure at the apex, and the lack of metatibial armature apart from a basal spine.

#### 3.2.3. Description of a New Species of *Cretodorus* with an Exceptionally Long Labium

Suborder Auchenorrhyncha Duméril, 1805

Infraorder Fulgoromorpha Evans, 1946

Superfamily Fulgoroidea Kirkaldy, 1907

Family Neazoniidae Szwedo, 2007 (=Mimarachnidae Shcherbakov, 2007 syn. nov.)

Genus *Cretodorus* Fu & Huang, 2020

Type species *Cretodorus granulatus* Fu & Huang, 2020

*Cretodorus noonoo* sp. nov. Fabrikant, Fu & Davranoglou

urn:lsid:zoobank.org:act:3CCB5BAB-F425-40D9-B432-986CA99DF1D0

Etymology. The new species is named after the anthropomorphic vacuum cleaner character “Noo-Noo” appearing in the popular kids TV-show Teletubbies in reference to its similarly elongated and flexible mouthparts.

Diagnosis. *C. noonoo* can be distinguished from all other congeneric species by the following combination of characters: tegmen length-width ratio 4.0 (others max 3.8), median carinae of mesonotum slightly converging anteriorly, wing length 7.2 mm, radial cell about two times wider than median cell, 4:4:4 metatibial metatarsal spination formula (other species of *Cretodorus* with 5 or 6 spines).

Description. Adult male (Holotype NIGP210707) Body, 8.7 mm long from snout to tip of abdomen. Nymph (Paratype NIGP210716) 4.8 mm long.

Head. Subtriangular in dorsal view; vertex with median and lateral carinae; eyes large, exceeding half of head length; 3–4 sensory pits on frontal apex; clypeus elongate, reaching mesocoxae; labium three segmented, extremely elongated, reaching twice the body length; tip of labium missing; labium segment lengths from proximal to distal: 0.36 mm/1.1 mm/9.6 mm; third labium segment throughout its length densely covered in transverse corrugations.

Thorax. Pronotum mottled, sub triangular with anteriorly slightly converging double median carinae, central disc elevated and protruding anteriad, lateral lobes projecting backwards forming a concave posterior margin; mesonotum sub-rhomboid with double median carinae slightly converging posteriad and lateral carinae diverging posteriad then converging near posterior margin; mesonotum posteriorly with cluster of three sensory pits placed between median and lateral carinae.

Legs. Legs slender and slightly setose; segment lengths (femur/tibia/tarsus): prothoracic leg 1.7 mm/1.6 mm/0.57 mm; segment lengths of mesothoracic leg 1.5 mm/2.0 mm/0.5 mm; protarsus and mesotarsus two-segmented and bearing prominent hooked claws; segment length of metathoracic leg 1.4 mm/2.5 mm/2.0 mm; metatibial pecten asetigerous, armed with 4 spines; metatarsomeres each with 4 spines.

Wings. Tegmina 7.2 mm long, 1.8 mm wide, about 4 times length to width ratio; coloration mottled; surface covered in a uniform polygonal meshwork of delicate veinlets; venation simplified, all veins parallel; stem Subcostal posterior + Radial + Medial vein (Scp + R + M) about half as long as stem ScP+R; Subcostal posterior + Radial anterior vein (ScP + Ra) and Radial posterior vein (RP) simple, slightly arched anteriad; Medial posterior vein (MP) simple, straight; Cubitus anterior vein (CuA) forked at about half tegmen length, CuA1 and CuA2 converging slightly near apex; Cubitus posterior vein (CuP) simple, straight; claval fracture not reaching wing tip; claval apex almost reaching wing tip; Postcubital (Pcu) and anal (A1) veins fused at a point slightly basad of CuA fork; Radial cell about two times wider than median cell. Hind wing hyaline, slightly shorter than tegmen, covered in a uniform meshwork of delicate veinlets forming larger polygonal cells than tegmen; costal margin proximally straight and distally arched at an angle; ScP and MP simple straight; CuA forking once distally; CuP and Pcu curving posteriad; multiple incompletely preserved anal veins. Abdomen flattened; male genitalia distinctly elongated; anal tube apically forked, anal styles oval. 

### 3.3. Short Descriptions of Additional Examined Specimens

The fossil planthopper nymphs and adults investigated in this study can be clearly attributed to Mimarachnidae by their flattened body, presence of sensory pits in small clusters of 2–3, sensory pits arranged in pairs on the lateral margins of the abdominal tergites, labium with specialized corrugated distal segment, and metatibiae with reduced armature (see amended diagnosis).

1. Specimen NIGP210709: A well-preserved nymph; body sub oval, 4.7 mm long; head small, labium extending beyond apex of the abdomen for about a third of body length.
2. Specimen NIGP210710: Exceptionally well-preserved nymph; body sub oval, 5.1 mm long; head small, labium extending beyond apex of abdomen for short distance in between finger-like processes of the IX sternite.
3. Specimen NIGP210711: A well-preserved nymph exuvium with head capsule opened; body sub oval with constriction at base of abdomen, 6.9 mm long; head small, labium extending beyond apex of abdomen for a short distance.
4. Specimen NIGP210712: A well-preserved nymph, with left mesotibia missing; body wide, sub oval, and 8.6 mm long; head small, labium extending beyond apex of the abdomen for about a third of body length.
5. Specimen NIGP210713: A well-preserved nymph with right metatibia missing; body narrow, sub oval, and 5.3 mm long; head small, labium extending beyond the apex of the abdomen for about a third of body length.
6. Specimen NIGP210714: A well-preserved nymph with both protibiae partially missing, and vegetal syn-inclusion obscuring some of the ventral thorax; body sub oval, 4.1 mm long; head small, labium extending beyond apex of abdomen for about a third of body length.
7. Specimen NIGP210706: Mostly complete adult male about 11.6 mm long, in semi-opaque and bubble rich amber; ventral surface of head obscured by debris; labium extending beyond apex of abdomen for about a third of body length; pronotum and mesonotum with double median carinae and retain sensory pits; wing venation simplified. Precosta + Costal Posterior (Pc+CP), Subcostal (Sc), Radial anterior (RA), and Medial posterior (MP) veins simple. Cubitus anterior vein (CuA) forked, Cubitus posterior vein (CuP) simple, Postcubital vein (PCu) fused with anal vein (A1) before mid-length of tegmen; tegmen surface covered in a polygonal meshwork of fine veinlets.
  Remarks: Specimen NIGP210706 is attributed to the genus *Cretodorus* based on the adult retention of sensory pits and the characteristic simplified venation [30,53].
8. Specimen NIGP210708: Incomplete adult about 9.4 mm long; dorsal surface of body missing; labium extending beyond apex of abdomen for about a third of body length; wing venation simplified. Precosta + Costal Posterior (Pc + CP), Subcostal (Sc), and Radial anterior (RA) veins simple, Medial posterior (MP) and Cubitus anterior (CuA) veins forked, Cubitus posterior vein (CuP) simple, Postcubital vein (PCu) vein fused with Anal vein (A1) before mid-length of tegmen; tegmen surface covered in a polygonal meshwork of fine veinlets; Pcu and A1 fused at point slightly basad of CuA fork.
  Remarks: The venation of the specimen is most similar to the genus *Burmissus* Shcherbakov, 2017 and can be distinguished from other genera with comparable venation by the straight veins and smooth wing margin ([51] Figure 1 p. 2).
9. Specimen NIGP210705: Complete adult male about 7.6 mm long, in semi-opaque and bubble rich amber; ventral surface of head obscured by debris; labium extending beyond apex of abdomen for about a third of body length; pronotum and mesonotum with double median carinae; wing venation simplified. Precosta + Costal Posterior (Pc + CP), Subcostal (Sc), and Radial anterior (RA) veins simple, Medial posterior vein (MP), and Cubitus anterior (CuA) veins forked, Cubitus posterior vein (CuP) simple, Postcubital vein (PCu) fused with Anal vein (A) before mid-length of tegmen; tegmen surface covered in a polygonal meshwork of fine veinlet; leg morphology obscured.
  Remarks: The venation of the specimen is most similar to the genus *Burmissus* Shcherbakov, 2017 and can be distinguished from other genera with comparable venation by the straight veins and smooth wing margin ([51] Figure 1 p. 2).
10. Specimen NIGP210715: Incomplete adult specimen, partially obscured by cracks and opaque syninclusions; the head, most of pronotum, mesonotum, apex of wings, and forelegs missing; labium extending beyond apex of abdomen for about a third of body length; pronotum and mesonotum lacking sensory pits; wing venation at base of wings simplified; leg morphology obscured.
  Remarks: The fragmentary state of preservation of the specimen prevents specific or generic taxonomic assignment.
11. Specimen NIGP210717: Incomplete adult specimen, with the head, pronotum, anterior mesonotum, base of wings, and legs missing; labium extending slightly beyond apex of abdomen; mesonotum with double median carinae and retains sensory pits; wing venation simplified. Precosta + Costal Posterior (Pc + CP), Subcostal (Sc), Radial anterior (RA), and Medial posterior (MP) veins simple, cubitus anterior vein (CuA) forked, cubitus posterior vein (CuP) simple, Postcubital vein (PCu) fused with anal vein (A) before mid-length of tegmen; tegmen surface covered in a meshwork of fine veinlets; leg morphology obscured.
  Remarks: Specimen NIGP210717 is attributed to the genus *Cretodorus* based on the adult retention of sensory pits and the characteristic simplified venation [30,53].
12. Specimen OUMNH-MZ8: Complete adult specimen of extant Issidae indet, about 5.6 mm long.

## 4. Discussion

### 4.1. Corrugated Feeding Structures in Other Insects

Corrugated or annulated feeding structures are unknown among extant Hemiptera but occur convergently in several unrelated insect groups. In present-day Heteroptera, elongated cuticular feeding structures that undergo repeated flexion, such as portions of the labrum, often display a wrinkled or corrugated cuticle [54,55,56].

The highly flexible proboscis of Lepidoptera bears a striking, yet superficial resemblance to the labium of Neazoniidae (=Mimarachnidae, syn. nov.). In both cases, the structure is dorsoventrally flattened and possesses externally corrugated surfaces associated with repeated bending [57,58]. Nevertheless, the lepidopteran proboscis is anatomically more complex, consisting of serially interlocking sclerotized rings connected by flexible membranes instead of a sinusoid corrugate cuticle of uniform width [59].

Comparable morphologies also occur in several long-proboscid Mesozoic Mecoptera from Cretaceous deposits. These insects possess elongated cylindrical proboscides with corrugated external surfaces remarkably similar to those observed in Neazoniidae, despite their fundamentally different mouthpart construction [60,61]. We interpret the repeated evolution of corrugated feeding structures in unrelated insect groups as strongly suggestive of convergence toward similar mechanical solutions involving flexibility combined with resistance to collapse.

Among Hemiptera, the most comparable structure to the neazoniid labium occurs in the recently described Cretaceous psylloid family Miralidae Shcherbakov, 2020, whose members possess a corrugated second labial segment [62,63]. Ivanov et al. [62] interpreted the miralid labium as a telescoping structure composed of serially interlocking sclerotized rings that confer the capability of shortening during feeding and increasing lateral flexibility. However, examination of the published figures does not reveal clear evidence for fully circumferential interlocking rings or nested frusta (conical folds) comparable to those of flexible plastic drinking straws that would be required for telescoping or contracting [64]. Instead, the miralid labium displays a remarkably similar organization to that of Neazoniidae, including ventral and lateral corrugations combined with a smoother dorsal surface and an inflated distal portion. We therefore interpret these structures as more likely representing mechanically flexible feeding organs rather than telescoping systems.

Furthermore, a corrugated and apparently flexible labium is also present in an undescribed Cretaceous heteropteran of pentatomomorphan affinities (personal observation by Fabrikant D.). The repeated occurrence of such structures among unrelated Cretaceous Hemiptera, despite their near-complete absence in extant taxa, suggests that Mesozoic terrestrial ecosystems favored feeding strategies that are poorly represented in modern insect faunas.

### 4.2. Functional Significance of Corrugated Structures

Computed tomographic scanning demonstrates that the corrugated surface sculpture of the apical labial segment in Neazoniidae represents true sinusoidal folds of the cuticle rather than superficial ornamentation (Figure 4). The cuticle appears relatively uniform in thickness throughout the segment, indicating that the corrugation likely functioned mechanically rather than representing reinforcement through differential sclerotization alone. The annulated structure, together with the frequent preservation of the labium in curved positions, strongly suggests that the apical segment was flexible in life (Figure 2E,F; [36] Figure 1A p. 4).

The folding of a material into alternating ridges and grooves, commonly referred to as corrugation, is widely used in engineering because it increases resistance to buckling while maintaining flexibility [65]. Corrugated sheets display increased rigidity to forces acting perpendicular to the folds while remaining flexible parallel to them. Similar effects occur in corrugated cylindrical structures, where transverse folds improve resistance to axial and lateral deformation while reducing bending stiffness [66,67].

In addition to increasing rigidity in static structures, corrugation is highly effective in producing longitudinally flexible tubes that resist collapse and kink formation during bending [68,69]. The corrugations distribute the deformation across multiple folds, reducing localized stress concentrations and allowing tighter bending radii without structural failure [68,70].

Unlike smooth-walled cylinders, corrugated tubes maintain a more stable lumen during flexion because bending becomes partially decoupled from ovalization of the tube walls, thereby reducing the likelihood of buckling [71]. Such properties are especially advantageous in elongated tubular structures that contain delicate internal anatomical components, such as the stylet bundle enclosed within the labium.

Comparable structural principles occur repeatedly in biological systems. Corrugated shell constructions in mollusks increase resistance against crushing predators and hydrostatic pressure [72,73], whereas the annulated tracheae of vertebrates prevent airway collapse during neck flexion and inhalation [74]. Similar strategies therefore appear repeatedly where flexibility must be combined with structural stability.

The morphology of the neazoniid labium further suggests preferential dorsoventral bending. The apical segment is dorsoventrally compressed and possesses a smooth non-corrugated dorsal surface, both of which would increase lateral rigidity while allowing greater flexion within the sagittal plane. This interpretation is directly supported by the consistently dorsoventral flexion of the labium in all examined specimens where it is preserved in a bent state (Figure 2E,F).

The lateral regions of the labium also possess denser and deeper corrugations than the ventral surface, potentially reflecting accommodation of rotational strain generated during sagittal bending. Alternatively, the finer and deeper or more chevron-like corrugations on the lateral edges of the labium may have somewhat compensated for the dorsoventral flattening of the labium to accommodate side to side flexion by improving the corrugate folding of the lateral margins [57,75].

We find insufficient evidence that the corrugated labium functioned as a telescoping or bellows-like structure, as suggested by Ivanov et al. [62] for extinct psylloids with a labium similar to neazoniid planthoppers. Effective bellows generally require discrete rigid rings connected by highly flexible membranous regions capable of axial compression or a chevron corrugate [64,75]. In contrast, the neazoniidan labium consists of continuous sinusoidal folds with relatively homogeneous cuticle thickness. Furthermore, the smooth dorsal plate would resist axial contraction rather than facilitate it. The available evidence therefore supports a function related primarily to controlled flexibility and resistance to collapse.

### 4.3. Proposed Feeding Mechanism and Function

The posterior orientation of the fulgoromorphan labium presents a biomechanical challenge for insects possessing extremely elongated mouthparts. In extant planthoppers, feeding is achieved by positioning the body obliquely relative to the plant surface and flexing the labium until the apex contacts the substrate beneath the body [76]. In Neazoniidae, on the other hand, the labium frequently exceeds body length, sometimes surpassing it by more than twofold.

If operated as in extant Fulgoromorpha, the apex of the labium would intercept the substrate behind the insect during flexion, complicating feeding maneuvers and preventing optimal penetration angles. The corrugated distal segment of the labium in Neazoniidae is the longest of the three and is connected by an elbow-like articulation at the base, suggesting enhanced bending ability in the ventral and anterior directions (Figure 4D). However, the proportions of this segment indicate that body elevation alone would likely not have been sufficient to facilitate its rotation into an anterior feeding position.

**Figure 6:**
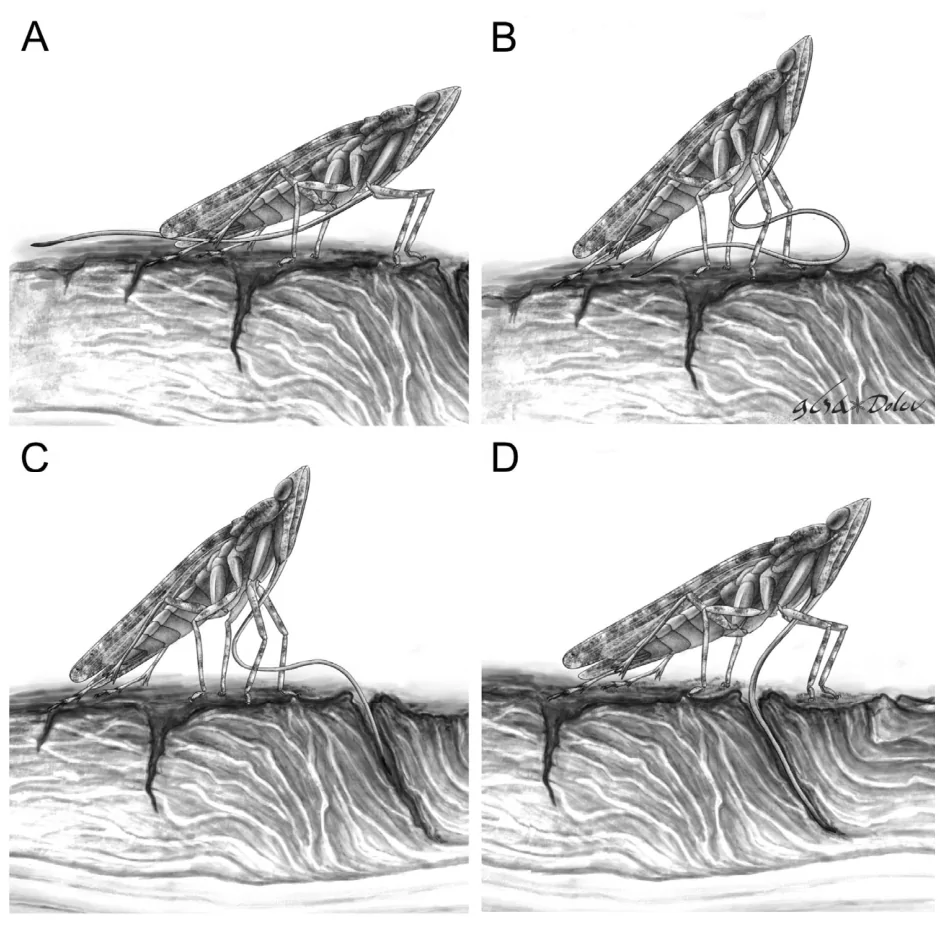
Artistic reconstruction of a speculative feeding sequence illustrating manipulation of the elongated labium in Neazoniidae. (**A**) Resting position with the labium extending posteriad beyond the hind margin of the insect (**B**) Process of bringing the labium forward via flexion of the elbow-like articulation at the base of the third segment and simultaneous bending of said segment (**C**) Initial probing of the surface of the bark and insertion of the labium into crevices (**D**) Labium in feeding position, fully inserted into the bark crevice to reach phloem. Artwork by Dolev Fabrikant.

The solution to this biomechanical constraint likely lies in the flexibility of this corrugated segment, which rather than behaving as a rigid rod, could have flexed dorsoventrally while being brought forward from rest into the feeding position (Figure 6A,B). Such flexibility would allow the structure to slide across the substrate in a curved configuration until the apex reached the desired feeding site, even in the confined space between the insect and the substrate (Figure 6B,C). This interpretation is particularly consistent with feeding within bark fissures or crevices. A flexible labium could potentially penetrate narrow irregular crevices inaccessible to rigid mouthparts and subsequently guide the stylets towards deeper vascular tissues (Figure 6D).

Similar ecological strategies occur in the extant aphid genus *Stomaphis*, which feeds through bark using highly elongated and flexible mouthparts that also exceed its body length [38]. The second segment of these aphids is membranous and possesses transverse reinforcing bars in a construction reminiscent but not directly comparable to those found in Neazoniidae. The first and second segments are connected by an articulation that allows the aphids to insert the labium into bark fissures while maintaining the base of the labium at a horizontal orientation during feeding. Interestingly, these insects solved the problem of directing the elongated labium by retracting the first two segments into the body cavity, thus shortening the labium into a manageable size. There is no evidence that neazoniids could do this due to the absence of telescoping adaptations such as a marked desclerotization of the labium.

The mechanism by which the flexible distal segment in Neazoniidae was controlled remains uncertain as it does not seem to have been retractable as in *Stomaphis* aphids. As extant Fulgoromorpha lack musculature within labial segment III, it is therefore possible that movement was generated proximally and transmitted along the corrugated distal segment. Steering may additionally have been aided by interactions between the labium and surrounding substrate surfaces, allowing bark crevices to function as external pivot points and guide channels.

Although internal steering by the stylets cannot be entirely excluded, several observations argue against this interpretation [77]. The stylet bundle is comparatively slender relative to the labial lumen and lacks obvious structural modifications that would facilitate forceful directional control. Furthermore, no clear pivot-like interlocking mechanisms were identified in the available micro-CT scans. The dorsoventral flattening of the labium would also mechanically hinder extensive lateral steering generated internally by the stylets.

The bilobed ribbed structure protruding at the apex of the labium may have functioned as an anchoring device during feeding. In extant planthoppers and other sap-feeding auchenorrhynchans, the mandibular stylets possess apical serrations that both stabilize the mouthparts and aid in the penetration of tough plant tissues and facilitate the deeper insertion of the sharper maxillary stylets into the plant vascular system [78,79]. The bilobed ribbed apical structures in Neazoniidae closely resemble the mandibular serrations in some extant auchenorrhynchans and may have similarly stabilized the feeding apparatus against mechanically resistant substrates such as bark ([80] Figure 16.1 p. 530).

**Figure 7:**
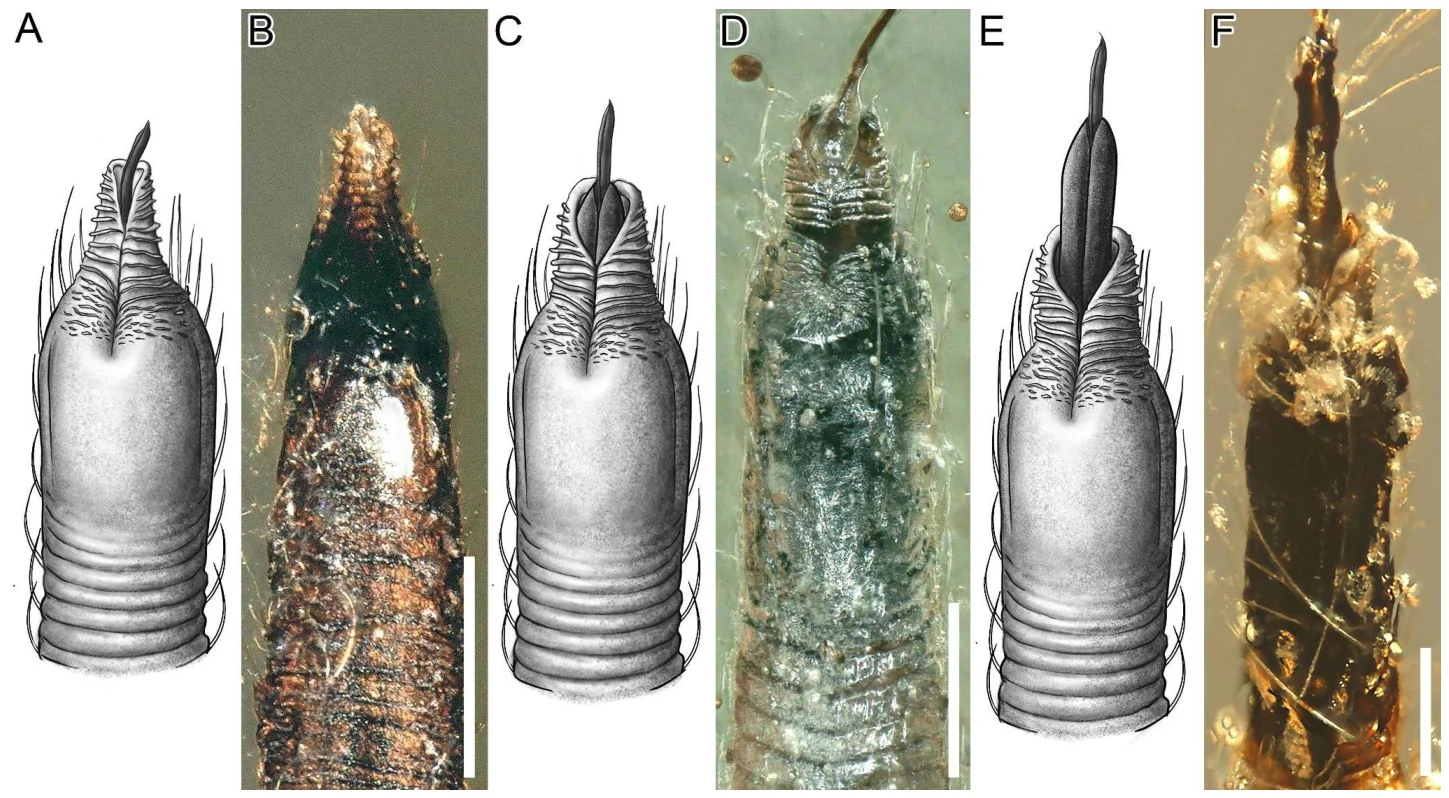
Observed conformations of the modified labium tip as a response to stylet bundle extension in ventral view. Scale bars: 100 μm. (**A**) Artistic reconstruction, tip structure mostly closed remaining narrow, maxillary stylets begin emerging. (**B**) Light photograph of specimen (NIGP210710) in the same conformation. (**C**) Artistic reconstruction, tip structure widening as the mandibular stylets pass through and begin emerging. (**D**) Light photograph of specimen (NIGP210706) in the same conformation. (**E**) Artistic reconstruction, tip structure fully expanded as stylets emerge fully. (**F**) Light photograph of specimen (NIGP210717) in the same conformation.

This protruding structure appears to have been narrower than the complete stylet bundle (Figure 1E). Considering the dimensions, the simultaneous emergence of the mandibular and maxillary stylets during feeding would have forced it to expand and the lateral ribbed lobes to open and anchor the labium to fissures in the surface, similar to a Screw Dübel in which the perimeter expands from the outward pressure applied by the screw. The pliability of the protruding ribbed structure is confirmed through its varied preservation in both open and closed configurations (Figure 7). To our knowledge, this structure and mode of mouthpart stabilization is unmatched in Hemiptera and may have facilitated stylet penetration by compensating for the reduced mechanical stability of the flexible labium, which likely could not provide as reliable an anchor through direct pressure as a straight, rigid labium.

### 4.4. Paleoecological and Evolutionary Implications

It may be tempting to suggest the possibility of nectarivory or pollination ecology for Neazoniidae because they coexisted with long-proboscid pollinating insects such as the butterfly-like kalligrammatid lacewings and siphonate Mecoptera during the rise of angiosperms [81]. Some Cretaceous mecopterans even possess corrugated proboscides superficially reminiscent of the mimarachnid labium [60,61]. Nevertheless, the available morphological evidence does not support a nectivorous ecology for these planthoppers.

Nectar-feeding insects generally require highly precise and repeatedly controlled mouthpart movements during probing of floral structures. Such mouthparts are typically directed anteriorly and optimized for repeated insertion and withdrawal. In contrast, the posteriorly directed and extremely elongated labium of Neazoniidae would likely have complicated repeated nectar-feeding maneuvers. The morphology of the feeding apparatus strongly supports an association with mechanically resistant plant substrates, most plausibly bark or deep vascular tissues of woody plants, rather than with repeated visitation of reproductive plant structure.

Furthermore, despite the abundance of neazoniid specimens in Burmese amber, no convincing examples preserve substantial pollen accumulations associated with the body. Previous studies attempting to infer pollination in fossil insects have emphasized the importance of both direct evidence, such as associated pollen, and specialized morphological adaptations facilitating nectar feeding [82]. At present, neither category of evidence is convincingly satisfied in Neazoniidae. Many neazoniids possess cryptic mottled coloration patterns and flattened body forms that suggest prolonged dwelling on exposed bark surfaces. Some taxa even display structural modifications interpreted as adaptations for visually disrupting the body outline [35,36,83,84]. Previous authors argued that mottled coloration and flattening indicate that Neazoniidae may have lived beneath tree bark and fed on fungal mycelia like some achilid nymphs [28].

However, the achilid nymphs generally lack the cryptic adaptations characteristic of neazoniid planthoppers and extraordinary structure of the elongated labium remain difficult to explain under a general fungal-feeding interpretation. It’s thus more likely that Neazoniidae were sap feeding bark specialists, using their elongated labium to probe deep surface fissures akin to the extant bark feeding aphids of the genus *Stomaphis* [38].

The variation in labium length among neazoniid taxa may reflect ecological partitioning rather than simple scaling with body size (Figure 1). Some taxa, such as *Cretodorus noonoo* sp. nov., possess extremely elongated mouthparts extending far beyond the abdominal apex, whereas others, including *Mimaeurypterus burmiticus* Fu & Huang, 2021 and *Multistria irregularis* Fabrikant & Fu, 2024, possess comparatively shorter labia [83,85,86]. Such variation may indicate specialization on different substrates, bark thicknesses, or host plants, potentially contributing to the remarkable diversity of Neazoniidae in Cretaceous ecosystems. The conservation of the corrugated construction despite variation in length may indicate a particular advantage to flexible feeding structures rather than just elongation, as suggested by previous authors [27,28,32].

The broad temporal range spanning 45 Ma, their wide geographic distribution across Eurasia and the repeated occurrence of corrugated feeding structures among unrelated Cretaceous Hemiptera further supports the stability and extent of Cretaceous resiniferous forest ecosystems [27,37,87,88]. The coniferous resin producing forests of the Cretaceous, including the Kachin amber forest, have been suggested to have experienced recurrent wildfires as evidenced by direct association with charcoal, with the amber deposits possibly being linked to massive resin production as a response to fire injury [88]. Trees growing in fire prone habitats show several adaptations for surviving wildfires, with the principal one being increased bark thickness [89]. The thicker bark of trees in fire prone Cretaceous ecosystems, and the persistence of gymnosperm-rich habitats during the Mesozoic Terrestrial Revolution may have provided ecological opportunities favoring elongated and flexible crevice-probing mouthparts supporting feeding strategies no longer represented among modern taxa [90].

## 5. Conclusions

In this study we describe a highly specialized corrugated labium in the Cretaceous planthopper family Neazoniidae. From a detailed analysis of the morphology, we infer the mechanical properties of the labium and suggest that the elongate structure was flexible and adapted for probing within tight spaces, likely fissures or cracks in bark or other resistant plant surfaces. Our findings indicate that Cretaceous planthoppers evolved feeding strategies absent from modern Auchenorrhyncha, highlighting a previously unrecognized component of Mesozoic ecological diversity. The repeated occurrence of long and corrugated feeding structures among unrelated Cretaceous insects suggests that the gymnosperm-dominated resiniferous forests of the Mesozoic supported specialized feeding strategies that lack close analogues among modern Hemiptera.

## Data Availability

The raw micro-computed tomography data supporting the conclusions of this article are part of ongoing research and will be made available by the authors on request.

## References

[1] Yoder J.B., Clancey E., Des Roches S., Eastman J.M., Gentry L., Godsoe W., Hagey T.J., Jochimsen D., Oswald B.P., Robertson J. (2010). Ecological opportunity and the origin of adaptive radiations. J. Evol. Biol..

[2] Friedrich F., Matsumura Y., Pohl H., Bai M., Hörnschemeyer T., Beutel R.G. (2014). Insect morphology in the age of phylogenomics: Innovative techniques and its future role in systematics. Entomol. Sci..

[3] Drohojowska J., Szwedo J., Żyła D., Huang D.-Y., Müller P. (2020). Fossils reshape the Sternorrhyncha evolutionary tree (Insecta, Hemiptera). Sci. Rep..

[4] Schuh R.T., Slater J.A. (1995). True Bugs of the World (Hemiptera: Heteroptera): Classification and Natural History.

[5] Schaefer C.W., Panizzi A.R. (2000). Heteroptera of Economic Importance.

[6] Johnson K.P., Dietrich C.H., Friedrich F., Beutel R.G., Wipfler B., Peters R.S., Allen J.M., Petersen M., Donath A., Walden K.K. (2018). Phylogenomics and the evolution of hemipteroid insects. Proc. Natl. Acad. Sci. USA.

[7] Misof B., Liu S., Meusemann K., Peters R.S., Donath A., Mayer C., Frandsen P.B., Ware J., Flouri T., Beutel R.G. (2014). Phylogenomics resolves the timing and pattern of insect evolution. Science.

[8] Song N., Wang M.-M., Huang W.-C., Wu Z.-Y., Shao R., Yin X.-M. (2024). Phylogeny and evolution of hemipteran insects based on expanded genomic and transcriptomic data. BMC Biol..

[9] Beutel R.G., Friedrich F., Yang X.-K., Ge S.-Q. (2013). Insect Morphology and Phylogeny: A Textbook for Students of Entomology.

[10] Boderau M., Nel A., Jouault C. (2025). Diversification and extinction of Hemiptera in deep time. Commun. Biol..

[11] Krenn H.W. (2019). Insect Mouthparts: Form, Function, Development and Performance.

[12] Spangenberg R., Wipfler B., Friedemann K., Pohl H., Weirauch C., Hartung V., Beutel R.G. (2013). The cephalic morphology of the Gondwanan key taxon *Hackeriella* (Coleorrhyncha, Hemiptera). Arthropod Struct. Dev..

[13] Grimaldi D., Engel M.S. (2005). Evolution of the Insects.

[14] Labandeira C.C., Phillips T.L. (1996). Insect Fluid-Feeding on Upper Pennsylvanian Tree Ferns (Palaeodictyoptera, Marattiales) and the Early History of the Piercing-and-Sucking Functional Feeding Group. Ann. Entomol. Soc. Am..

[15] Dmitriev D.A., Angelova R., Anufriev G.A., Bartlett C.R., Blanco-Rodríguez E., Borodin O.I., Cao Y.H., Cara C., Deitz L.L., Dietrich C.H. (2022). Fulgoromorpha Evans, 1946. World Auchenorrhyncha Database. https://hoppers.speciesfile.org/otus/56032/overview.

[16] Deng J., Stroiński A., Szwedo J., Ghanavi H.R., Yapar E., Franco D.C., Prus-Frankowska M., Michalik A., Wahlberg N., Łukasik P. (2025). Phylogenomic insights into the relationship and the evolutionary history of planthoppers (Insecta: Hemiptera: Fulgoromorpha). Syst. Entomol..

[17] Wilson S.W. (2005). Keys to the Families of Fulgoromorpha with Emphasis on Planthoppers of Potential Economic Importance in the Southeastern United States (Hemiptera: Auchenorrhyncha). Fla. Entomol..

[18] Bräunig P., Krumpholz K., Baumgartner W. (2012). Sensory pits—Enigmatic sense organs of the nymphs of the planthopper *Issus coleoptratus* (Auchenorrhyncha, Fulgoromorpha). Arthropod Struct. Dev..

[19] Davranoglou L.-R., Cicirello A., Taylor G.K., Mortimer B. (2019). Planthopper bugs use a fast, cyclic elastic recoil mechanism for effective vibrational communication at small body size. PLoS Biol..

[20] O’Brien L.R., Wilson S.W., Nault L.R., Rodriguez J.G. (1985). Planthopper Systematics and External Morphology. The Leafhoppers and Planthoppers.

[21] Shcherbakov D.E., Popov Y.A., Rasnitsyn A.P., Quicke D.L.J. (2002). Superorder Cimicidea Laicharting, 1781 Order Hemiptera Linné, 1758. The Bugs, Cicadas, Plantlice, Scale Insects, Etc.(= Cimicida Laicharting, 1781,= Homoptera Leach, 1815+ Heteroptera Latreille, 1810). History of Insects.

[22] Attié M., Bourgoin T., Veslot J., Soulier-Perkins A. (2008). Patterns of trophic relationships between planthoppers (Hemiptera: Fulgoromorpha) and their host plants on the Mascarene Islands. J. Nat. Hist..

[23] Cook A.G., Denno R.F., Denno R.F., Perfect T.J. (1994). Planthopper/Plant Interactions: Feeding Behavior, Plant Nutrition, Plant Defense, and Host Plant Specialization. Planthoppers: Their Ecology and Management.

[24] Strong D.R., Lawton J.H., Sir Southwood R. (1984). Insects on Plants: Community Patterns and Mechanisms.

[25] Benton M.J., Wilf P., Sauquet H. (2022). The Angiosperm Terrestrial Revolution and the Origins of Modern Biodiversity. New Phytol..

[26] Haug C., Herrera-Flórez A.F., Müller P., Haug J.T. (2019). Cretaceous chimera–an unusual 100-million-year old neuropteran larva from the “experimental phase” of insect evolution. Palaeodiversity.

[27] Szwedo J. (2007). Nymphs of a new family Neazoniidae fam. n. (Hemiptera: Fulgoromorpha: Fulgoroidea) from the Lower Cretaceous Lebanese amber. Afr. Invertebr..

[28] Szwedo J. (2009). First discovery of Neazoniidae (Insecta, Hemiptera, Fulgoromorpha) in the Early Cretaceous amber of Archingeay, SW France. Geodiversitas.

[29] Shcherbakov D.E. (2007). Mesozoic Spider Mimics-Cretaceous Mimarachnidae fam.n. (Homoptera: Fulgoroidea). Russ. Entomol. J..

[30] Liu X.-J., Jiang H., Chen J., Fu Y.-Z., Boderau M., Bojarski B., Bruthansová J., Nyunt T.T., Szwedo J. (2024). Investigation of *Cretodorus* (Hemiptera: Fulgoromorpha: Mimarachnidae) based on new adult and nymph fossils from mid-Cretaceous Myanmar amber. Mesozoic.

[31] Wang W., Zhuo D., Zheng Y., Cui X., Luo C., Chen J. (2025). A New Genus and Two New Species of Planthopper (Hemiptera, Fulgoromorpha, Mimarachnidae) from the Mid-Cretaceous Kachin Amber of Myanmar. Alcheringa Australas. J. Palaeontol..

[32] Amaral A.P., Stotzem L., Haug G.T., Haug C., Haug J.T. (2024). Immature planthoppers had longer mouthparts 100 million years ago as exemplified by quantitative morphology. Spixiana.

[33] Brysz A.M., Szwedo J. (2019). Jeweled Achilidae—A new look at their systematics and relation to other Fulgoroidea (Hemiptera). Monogr. Up. Silesian Mus..

[34] Kiesmüller C., Haug G.T., Haug C., Müller P., Hörnig M.K., Haug J.T. (2024). New indications for the life habits of long-legged aphidlion-like larvae in about 100-million-year-old amber. PalZ.

[35] Haug J.T., Fu Y., Haug G.T., Haug C. (2026). Disruptive colouration is much more common in immature animals from the Myanmar amber forest than anticipated, indicating regular convergent evolution. Mesozoic.

[36] Luo C., Wang B., Jarzembowski E.A. (2021). A Bizarre Planthopper Nymph (Hemiptera: Fulgoroidea) from Mid-Cretaceous Kachin Amber. Insects.

[37] Szwedo J., Ansorge J. (2015). The first Mimarachnidae (Hemiptera: Fulgoromorpha) from Lower Cretaceous lithographic limestones of the Sierra Del Montsec in Spain. Cretac. Res..

[38] Brożek J., Mróz E., Wylężek D., Depa Ł., Węgierek P. (2015). The structure of exetremely long mouthparts in the aphid genus *Stomaphis* Walker (Hemiptera: Sternorrhyncha: Aphididae). Zoomorphology.

[39] Li X., Tian L., Li H., Cai W. (2021). Ultrastructural Variations of Antennae and Labia Are Associated with Feeding Habit Shifts in Stink Bugs (Heteroptera: Pentatomidae). Biology.

[40] Sutton M.F. (1951). On the food, feeding mechanism and alimentary canal of Corixidae (Hemiptera, Heteroptera). Proc. Zool. Soc. Lond..

[41] Wang Y., Zhang J., Wang W., Brożek J., Dai W. (2020). Unique Fine Morphology of Mouthparts in *Haematoloecha nigrorufa* (Stål) (Hemiptera: Reduviidae) Adapted to Millipede Feeding. Insects.

[42] Bourgoin T., Wang R.-R., Asche M., Hoch H., Soulier-Perkins A., Stroiński A., Yap S., Szwedo J. (2015). From micropterism to hyperpterism: Recognition strategy and standardized homology-driven terminology of the forewing venation patterns in planthoppers (Hemiptera: Fulgoromorpha). Zoomorphology.

[43] Wilde F., Ogurreck M., Greving I., Hammel J.U., Beckmann F., Hipp A., Lottermoser L., Khokhriakov I., Lytaev P., Dose T. (2016). Micro-CT at the imaging beamline P05 at PETRA III. AIP Conf. Proc..

[44] Gorman M.J., Arakane Y. (2010). Tyrosine hydroxylase is required for cuticle sclerotization and pigmentation in *Tribolium castaneum*. Insect Biochem. Mol. Biol..

[45] Brożek J., Bourgoin T., Szwedo J. (2006). The interlocking mechanism of maxillae and mandibles in fulgoroidea (Insecta: Hemiptera: Fulgoromorpha). Pol. J. Entomol..

[46] Emeljanov A.F. (2001). Larval characters and their ontogenetic development in Fulgoroidea (Homoptera, Cicadina). Zoosyst. Ross..

[47] Gnezdilov V.M., Wilson M.R. (2007). A new genus and a new species of the tribe Mithymnini (Hemiptera: Fulgoromorpha: Nogodinidae) from Namibia, with sternal sensory pits in the adult. Zootaxa.

[48] Gnezdilov V.M., Holzinger W.E., Wilson M.R. (2014). The Western Palaearctic Issidae (Hemiptera, Fulgoroidea): An Illustrated Checklist and Key to Genera and Subgenera. Proc. Zool. Inst. RAS.

[49] Zhang H., Cai J.-H., Qin D.-Z. (2024). Fine morphology of eggs, nymphs, wax-secreting structures and sensory pits of the planthopper *Euricania clara* Kato (Hemiptera: Fulgoromorpha: Ricaniidae), with comparative notes on related species. Zoomorphology.

[50] Gnezdilov V.M., Wilson M.R. (2006). Systematic notes on tribes in the family Caliscelidae (Hemiptera: Fulgoroidea) with the description of new taxa from Palaearctic and Oriental Regions. Zootaxa.

[51] He J., Jiang T., Guan X., Szwedo J. (2022). A new genus and species of family Mimarachnidae (Hemiptera: Fulgoromorpha: Fulgoroidae) from mid-Cretaceous Kachin amber, northern Myanmar. Cretac. Res..

[52] Shcherbakov D.E. (2017). First record of the Cretaceous family Mimarachnidae (Homoptera: Fulgoroidea) in amber. Russ. Entomol. J..

[53] Fu Y., Huang D. (2020). New data on fossil mimarachnids (Hemiptera, Fulgoromorpha, Fulgoroidea) in mid-Cretaceous Burmese amber. Palaeoentomology.

[54] Taszakowski A., Masłowski A., Brożek J. (2022). Labial Sensory Organs of Two *Leptoglossus* Species (Hemiptera: Coreidae): Their Morphology and Supposed Function. Insects.

[55] Wang Y., Dai W. (2020). How Does the Intricate Mouthpart Apparatus Coordinate for Feeding in the Hemimetabolous Insect Pest *Erthesina fullo*?. Insects.

[56] Shi Y., Depa Ł., Brożek J., Dai W. (2026). Morphological Modification of the Mouthparts of Aphids (Hemiptera: Sternorryncha: Aphididae). Insects.

[57] Chen D., Song H., Liu Q., Gan J., Liu Y., Chen K., Wang C., Wen S., Zhou Y., Yan C. (2020). Large Curvature Folding Strategies of Butterfly Proboscis. J. Bionic Eng..

[58] Yoshioka T., Stepanova T., Brasovs A., Blouin V.Y., Beard C.E., Adler P.H., Kornev K.G. (2025). The crescent cross-section and dichotomous chitin structure make the proboscis of butterflies and moths a hydraulic spring. Acta Biomater..

[59] Stepanova T. (2020). Characterization of Mechanical, Thermodynamic and Surface Properties of the Butterfly Proboscis. Master’s Thesis.

[60] Ren D., Labandeira C.C., Santiago-Blay J.A., Rasnitsyn A., Shih C., Bashkuev A., Logan M.A.V., Hotton C.L., Dilcher D. (2009). A Probable Pollination Mode before Angiosperms: Eurasian, Long-Proboscid Scorpionflies. Science.

[61] Zhao X., Wang B., Bashkuev A.S., Aria C., Zhang Q., Zhang H., Tang W., Engel M.S. (2020). Mouthpart homologies and life habits of Mesozoic long-proboscid scorpionflies. Sci. Adv..

[62] Ivanov G.A., Vorontsov D.D., Shcherbakov D.E. (2025). A remarkable psyllomorph family from Cretaceous Burmese amber, Miralidae stat. nov.(= Dinglidae syn. nov.; Hemiptera: Sternorrhyncha). Cretac. Res..

[63] Shcherbakov D.E. (2020). New Homoptera from the Early Cretaceous of Buryatia with notes on the insect fauna of Khasurty. Russ. Entomol. J..

[64] Wo Z., Filipov E.T. (2022). Bending Stability of Corrugated Tubes with Anisotropic Frustum Shells. J. Appl. Mech..

[65] Garbowski T., Knitter-Piątkowska A. (2022). Analytical Determination of the Bending Stiffness of a Five-Layer Corrugated Cardboard with Imperfections. Materials.

[66] Ghazijahani T.G., Dizaji H.S., Nozohor J., Zirakian T. (2015). Experiments on corrugated thin cylindrical shells under uniform external pressure. Ocean. Eng..

[67] Norman A.D., Seffen K.A., Guest S.D. (2009). Morphing of curved corrugated shells. Int. J. Solids Struct..

[68] Riva M., Airoldi A., Turconi T., Ballarin P., Boiocchi M., Bottasso L. (2023). Development and manufacturing of flexible joints based on corrugated composite laminates. Compos. Struct..

[69] Robinson A., Duran J.S.H., Jiang D., Leung J., Laude M., Nkansah A., Guo L., Timmins L., Cosgriff-Hernandez E. (2025). Design of a Corrugated Vascular Graft with Enhanced Compliance and Kink Resistance. J. Biomed. Mater. Res. A.

[70] Wo Z., Filipov E.T. (2023). Stiffening multi-stable origami tubes by outward popping of creases. Extrem. Mech. Lett..

[71] Brazier L.G. (1927). On the flexure of thin cylindrical shells and other “thin” sections. Proc. R. Soc. Lond. Ser. Contain. Pap. Math. Phys. Character.

[72] Ward P. (1981). Shell Sculpture as a Defensive Adaptation in Ammonoids. Paleobiology.

[73] Zuschin M., Stachowitsch M., Stanton R.J. (2003). Patterns and processes of shell fragmentation in modern and ancient marine environments. Earth-Sci. Rev..

[74] Safshekan F., Tafazzoli-Shadpour M., Abdouss M., Shadmehr M.B. (2016). Mechanical Characterization and Constitutive Modeling of Human Trachea: Age and Gender Dependency. Materials.

[75] Reid A., Lechenault F., Rica S., Adda-Bedia M. (2017). Geometry and design of origami bellows with tunable response. Phys. Rev. E.

[76] Hao Y., Dietrich C.H., Dai W. (2016). Structure and Sensilla of the Mouthparts of the Spotted Lanternfly *Lycorma delicatula* (Hemiptera: Fulgoromorpha: Fulgoridae), a Polyphagous Invasive Planthopper. PLoS ONE.

[77] Pollard D.G. (1969). Directional control of the stylets in phytophagous Hemiptera. Proc. R. Entomol. Soc. Lond. (A).

[78] Mora R., Retana A., Espinoza A.M. (2001). External Morphology of *Tagosodes orizicolus* (Homoptera: Delphacidae) Revealed by Scanning Electron Microscopy. Ann. Entomol. Soc. Am..

[79] Backus E.A., Nault J.R., Rodriguez J.G. (1985). Anatomical and sensory mechanisms of leafhopper and planthopper feeding behavior. The Leafhoppers and Planthoppers.

[80] Rakitov R., Krenn H.W. (2019). Morphogenesis of Piercing Stylets in Hemiptera. Insect Mouthparts: Form, Function, Development and Performance.

[81] Khramov A.V., Bashkuev A.S., Lukashevich E.D. (2020). The Fossil Record of Long-Proboscid Nectarivorous Insects. Entomol. Rev..

[82] Peña-Kairath C., Delclòs X., Álvarez-Parra S., Peñalver E., Engel M.S., Ollerton J., Peris D. (2023). Insect pollination in deep time. Trends Ecol. Evol..

[83] Fabrikant D., Huang D.-Y., Haug C., Haug J.T., Fu Y.-Z. (2024). Morphological features of the Upper Cretaceous *planthopper Mimaeurypterus burmiticus* suggest specialization for cryptic camouflage on tree bark. Mesozoic.

[84] Jiang T., Szwedo J., Wang B. (2019). A unique camouflaged mimarachnid planthopper from mid-Cretaceous Burmese amber. Sci. Rep..

[85] Fabrikant D., Huang D.-Y., Fu Y.-Z. (2024). Three new species of *Multistria* from mid-Cretaceous amber of northern Myanmar (Hemiptera: Fulgoromorpha: Mimarachnidae). Mesozoic.

[86] Fu Y., Huang D. (2021). New mimarachnids in mid-Cretaceous amber from northern Myanmar (Hemiptera, Fulgoromorpha). Cretac. Res..

[87] Barrón E., Peyrot D., Bueno-Cebollada C.A., Kvaček J., Álvarez-Parra S., Altolaguirre Y., Meléndez N. (2023). Biodiversity of ecosystems in an arid setting: The late Albian plant communities and associated biota from eastern Iberia. PLoS ONE.

[88] Delclòs X., Peñalver E., Barrón E., Peris D., Grimaldi D.A., Holz M., Labandeira C.C., Saupe E.E., Scotese C.R., Solórzano-Kraemer M.M. (2023). Amber and the Cretaceous Resinous Interval. Earth-Sci. Rev..

[89] Schafer J.L., Breslow B.P., Hohmann M.G., Hoffmann W.A. (2015). Relative Bark Thickness Is Correlated with Tree Species Distributions along a Fire Frequency Gradient. Fire Ecol..

[90] Lidgard S., Crane P.R. (1990). Angiosperm Diversification and Cretaceous Floristic Trends: A Comparison of Palynofloras and Leaf Macrofloras. Paleobiology.

